# Editorial: The development and evolution of larval nervous systems

**DOI:** 10.3389/fnins.2025.1721513

**Published:** 2025-11-17

**Authors:** Oliver Vöcking, Allan Carrillo-Baltodano, Néva P. Meyer

**Affiliations:** 1 Department of Molecular and Cellular Biochemistry, University of Kentucky, Lexington, KY, United States; 2 School of Biological and Behavioral Sciences, Queen Mary University London, London, United Kingdom; 3 Department of Biology, Clark University, Worcester, MA, United States

**Keywords:** neuron, invertebrates, larva, development, serotonin

Throughout their development, animals of various taxa go through different larval stages, which have evolved adaptations to interact with specific environments. Larval nervous systems in particular experience strong selection pressures since they control crucial physiological and behavioral processes, especially in taxa with planktotrophic larvae that need to acquire food. Selection pressures also can vary across life history stages if there are differences in factors such as habitat, predation avoidance, and diet. There is evidence for this in the relationship of the larval nervous system to the adult nervous system, which can vary widely between species. Understanding the specific adaptations of larval nervous systems requires an understanding of basic developmental processes such as cell migration, proliferation, and differentiation. In several species, the larval stages are still understudied and many questions about the development, physiology, and morphology of their nervous systems remain unanswered. Furthermore, comparisons of larval nervous systems among different animal species can be highly useful for reconstructing how the nervous systems evolved under similar or different selection pressures.

The studies in this Research Topic represent the great diversity of animal larval types including species of Cnidaria, Annelida, Mollusca, Echinoidea, Nemertea and Insecta. These include the extensively studied organism *Drosophila melanogaster*, as well as organisms that are gaining in research popularity like the cnidarian *Cassiopea xamanachana* and the annelid *Malacoceros fuliginosus* as well as organisms that have hardly been studied such as the nemertean *Cephalorhix rufifrons*. Each of these studies will contribute to a better understanding of animal neuronal evolution and development.

## 1 Comparative neural anatomy

Kalke et al. use immunohistochemistry and confocal laser scanning microscopy to characterize the anterior nervous system with a focus on innervation of palp-like structures in the planktotrophic larvae of three phylogenetically-distinct species of annelids, *Magelona mirabilis* (Magelonidae), *Paramphinome jeffreysii* (Amphinomidae) and *Malacoceros fuliginosus* (Spionidae). They also employ azan-staining of histological sections of the head in adults of *Eurythoe complanata* (Amphinomidae) to generate 3D reconstructions of the anterior nervous system. The authors report similar innervation of palp-like structures in larvae and adults of Magelonidae and Spionidae, which is comparable to innervation of the feeding palps of other annelid species, suggesting homology of these structures across Annelida. In *P. jeffreysii* (Amphinomidae), innervation of the larval feeding palps also appears homologous with other annelid species, but this pattern of innervation was not observed for adult head appendages, including the antennae, in *E. complanata* (Amphinomidae), suggesting loss of the larval palps at metamorphosis. The authors propose that evolutionary loss of the larval palps in Amphinomidae may have accompanied the transition of feeding modes from a planktotrophic larvae to an omnivorous scavenging or carnivorous adult, making this group particularly interesting for future studies of sensory system evolution.

Another extraordinary example of body adaptations related to feeding is present in the annelid *Osedax japonicus*, a member of the commonly deep-sea inhabitants Siboglinidae. Species of *Osedax* have modifications of their body in the form of roots, that in combination with symbiotic bacteria enable them to obtain resources from decaying bone remains that fall into the deep-sea (Rouse et al., 2004, 2009). As if the feeding strategy was not surprising enough, *Osedax* also presents a strong dwarfism, with large females being populated by a harem of dwarf males. Worsaae et al. describe the neuromuscular systems of five developmental stages of *O. japonicus* to understand the developmental timepoint where these large anatomical differences first appear. They highlight the retention of several larval nervous system features after metamorphosis of males, and proposing paedomorphosis of males as a way to secure scarce substrates in the deep-sea environment.

von Döhren does a phylum wide comparison in Nemertea, by focusing on traditional neural markers during early nervous system development, such as serotonin, FMRFamide and Synapsin-like immunoreactivity in two ribbon worm species of the distinct clades *Cephalothrix rufifrons* (Archinemertea, Palaeonemertea) and *Emplectonema gracile* (Monostilifera, Hoplonemertea). The author describes first stages of neurogenesis, the subsequent formation of a ring-shaped brain, and discusses the benefit of using Synapsin-lir instead of the more traditional Tubulin labeling to assess the neurite architectures. Not only does this study hypothesize a ground pattern of nemertean neural development, but it also allows comparisons to other lophotrochozoan species.

A synergy of techniques has always been the key to understand both morphology and infer function of neurons (Williams and Jékely, 2016). Both Seybold et al. and Cocurullo et al. tackle the study of pioneering and neurosecretory neurons by combining *in situ* hybridization (ISH) and electron microscopy. In the annelid *M. fuliginosus*, Seybold et al. traced the development of specific neurons using serial block dissections, transmission electron microscopy and 3D reconstructions, and correlated them to *pkd*+ expressing cells, that in other systems like *Platynereis dumerilii* have been shown to be mechanosensory neurons (Bezares-Calderon et al., 2018). Therefore, these so-called pioneer neurons not only have a critical function for the larval nervous system, such as presumably controlling swimming behavior and sensing, but also set the foundation for the later developing adult nervous system. Cocurullo et al. cleverly identified presumable neurosecreting cells by utilizing scanning electron microscopy on freeze-fractured larvae. These cells are in the position where Thyrotropin markers are identified using immunohistochemistry and ISH in the pluteus. These cells were present during the development in different sea urchin species, in correlation with *opsin* expression, giving insights into evolutionary aspects of neuropeptide and photoreception in sea urchins.

An upcoming model organism in the form of the cnidarian species *Cassiopea xamachana* is studied by Amplatz et al.. The authors demonstrate that the larvae of the species develop a concentrated nerve net, which is subsequently replaced by an orally concentrated nerve net when the animals reach adulthood. Due to their findings and comparisons with other cnidarian species, the authors suggest that this kind of development may represent an ancestral trait in cnidarian evolution.

Two studies of the Research Topic chose different mollusk species as study organisms. Hasan et al. provide a detailed analysis of the protein family of opsins in different bivalve species. For their approach they used a combination of a phylogenetic and genomic approaches. Not only did they find great diversity of the different opsin families among bivalve mollusks, but also specific differences in expression patterns within the species. Their results corroborate previous analyses that larval mollusks express a great variety of opsins in different body regions. Further, they show that gene expression can increase according to the life stage. Kurtova et al. on the other hand analyzed neurogenesis in the gastropod *Lymnea stagnalis*. They focused on expression of the *soxB* transcription factors. While *sox* genes are known to be important for several developmental processes, their expression is largely unknown in many taxa. The authors found expression differences in *soxB1* and *soxB2*. While they found *soxB1* to be more broadly expressed in different tissues including the head, foot, visceral complex and developing sensory cells, *soxB2* was mostly limited to developing ganglia cells during metamorphosis. When compared to other lophotrochozoan species, the authors found that *soxB* gene expression is prolonged and more widespread, which they interpret as a form of transcriptional neoteny.

## 2 Novel approaches to study neural behavior

The article by Obukhova et al. compares nervous system plasticity between the planktotrophic, pluteus larvae of two sea urchin species, which is of particular interest for understanding adaptations. The authors scored 5HT+ and dopamine+ neurons in gastrula to pluteus stages of *Mesocentrotus nudus* and *Paracentrotus lividus* and found variation in the number of post-oral, dopamine+ neurons in prism and pluteus stage larvae. The authors also found that increased numbers of post-oral dopamine+ neurons or treatment with dopamine was correlated with downward swimming while increased numbers of 5HT+ neurons or treatment with 5HT was correlated with upward swimming. The authors propose that increasing the ratio of dopamine+ to 5HT+ neurons, both of which likely control ciliary beating, drives larvae closer to the benthos where they will metamorphose. The variation in numbers of dopamine+ neurons across individuals within a species over developmental time may underlie the observed variation in swimming behavior and could drive selectable variation in dispersal and time to settlement in both species. This illustrates how variation in number and timing of appearance of larval neurons could drive behavioral evolution of swimming behavior.

Lastly, Matos et al. present a novel approach to analyze neuromuscular network activities and compare them among different larval stages throughout development in *Drosophila*. They developed a mathematical method that uses a graph theory approach in combination with calcium staining to analyze neuronal activity waves. The application of this method allowed them to highlight the differences in neuronal activity patterns between L1 and L3 instar larvae. Their findings will not only be beneficial for invertebrate biology, but have the potential to improve our understanding of motor control and neural coordination.

The articles in this Research Topic demonstrate the immense diversity in larval nervous systems and different solutions to environmental challenges by various species. The presented studies increase our understanding of the evolution and development of larval nervous systems, as well as set the foundation for future analyses to further deepen our knowledge.
